# Combination of tomographic ultrasound imaging and three-dimensional magnetic resonance imaging-based model to diagnose postpartum levator avulsion

**DOI:** 10.1038/s41598-017-08201-9

**Published:** 2017-09-11

**Authors:** Yulin Yan, Chaoran Dou, Xia Wang, Yan Xi, Bing Hu, Li Ma, Tao Ying

**Affiliations:** 1 Department of Ultrasound in Medicine, Shanghai Jiao Tong University Affiliated Sixth People’s Hospital, Shanghai, China; 2 Shanghai Institute of Ultrasound in Medicine, Shanghai, China; 3 Department of Obstetrics and Gynecology, Shanghai Jiao Tong University Affiliated Sixth People’s Hospital, Shanghai, China; 4 Department of Radiology, Shanghai Jiao Tong University Affiliated Sixth People’s Hospital, Shanghai, China

## Abstract

Vaginal delivery may cause levator avulsion, which may increase the risk of pelvic floor dysfunction (PFD). To explore the morphological changes of the levator ani muscle (including the puborectalis and iliococcygeus) and levator avulsion after vaginal delivery, translabial tomographic ultrasound imaging (TUI) was used to examine 80 women 45–60 days after their vaginal delivery. Subsequently, magnetic resonance imaging (MRI) was performed if at least one-sided puborectalis avulsion was found on TUI. The incidence of puborectalis avulsion in these postpartum women was 13.75% in this study. Both MRI and TUI can detect puborectalis avulsion well, and their results have good consistency. Iliococcygeus muscle injury is difficult to detect using TUI. However, MRI is a good way to observe the morphological changes of the iliococcygeus, which may also be damaged during vaginal delivery. Interestingly, our study reveals that iliococcygeus muscle injury is often associated with severe puborectalis muscle tear.

## Introduction

The levator ani muscle, mainly including the puborectalis and iliococcygeus, is considered to play a vital role in the maintenance of pelvic organ support and function^1^. Studies have shown that pelvic organ prolapse is closely related to levator avulsion. Women with levator avulsion are more likely to have pelvic organ prolapse than those without, and levator avulsion is associated with a recurrence of pelvic organ prolapse^2, 3^. Therefore, the exploration of these injuries, especially the complete avulsion of the levator ani muscle, has been a growing interest. Delivery-related traumas are the most important causes of levator avulsion^4^, especially the trauma caused by forceps^5^. Vaginal delivery may cause enlargement of the hiatus, particularly during the crowning of the foetal head, and lead to the avulsion of the levator ani muscle from the pubis^6^. Vaginal delivery may also cause macroscopic visible tear of the levator ani muscle, including the puborectalis and iliococcygeus, which can be diagnosed by imaging^7, 8^. According to a previous study, the avulsion of puborectalis from the pelvic sidewall occurred in 23% of vaginally parous women^8^.

Levator avulsion can be detected by palpation, magnetic resonance imaging (MRI) and translabial three-dimensional ultrasound imaging. Clinical digital palpation is a good method to detect levator avulsion but requires doctors to have substantial teaching and clinical experience, and there is limited repeatability. Currently, modern imaging methods can be a reliable way to diagnose levator avulsion^9, 10^. Translabial ultrasound is widely used and accepted for diagnosing levator avulsion. 3D volume rendering and tomographic ultrasound imaging (TUI) have the ability to visualize the axial plane with good spatial and superior temporal resolution^11–13^. Additionally, due to its exquisite soft tissue contrast and discriminatory capabilities, MRI is considered to be the most reliable reference standard and has been used to observe levator ani trauma since the 1990s^14, 15^. The components of the levator ani muscle can be clearly distinguished in MRI scans. Due to the complicated three-dimensional formation of levator ani muscle, two-dimensional (2D) magnetic resonance images cannot sufficiently demonstrate the relationship of the muscles. However, three-dimensional models can provide more sufficient and valid results with some reconstruction software^16^.

However, few published literatures have compared levator avulsion before and after vaginal delivery with the help of TUI and MRI. Therefore, this study was designed to observe the morphological changes of the levator ani muscle, detect levator avulsion after vaginal delivery using translabial 3D ultrasound and MRI and compare the consistency of ultrasound and MRI in diagnosing levator avulsion.

## Results

### Information

All the datasets could be used for analysis. Table 1 lists the demographic data of the two groups. The 80 vaginally parous women included 72 spontaneous first vaginal deliveries and 8 forceps deliveries. The interval was 50.66 ± 5.75 days after delivery (within 45–60 days). The age of the vaginally parous women was 24.32 ± 3.70 years old, and that of the nulliparous women was 23.84 ± 3.85 years old. The average gravidity of the vaginally parous women was 2.2 (range 1–3) (Table 1).

**Table 1:** General demographics of patients in the primiparae group and the nulliparae group.

Variables	Primiparae group	Nulliparae group
Age (y)	24.32 ± 3.70	23.84 ± 3.85
BMI (kg/m^2^)	23.08 ± 2.14	22.49 ± 1.53
Gravidity (n)	2.2 (1–3)	0
Parity (n)	1	0
Birth weight(g)	3278 ± 419 (2200–3940)	—

### Ultrasound results

**Figure 1:**
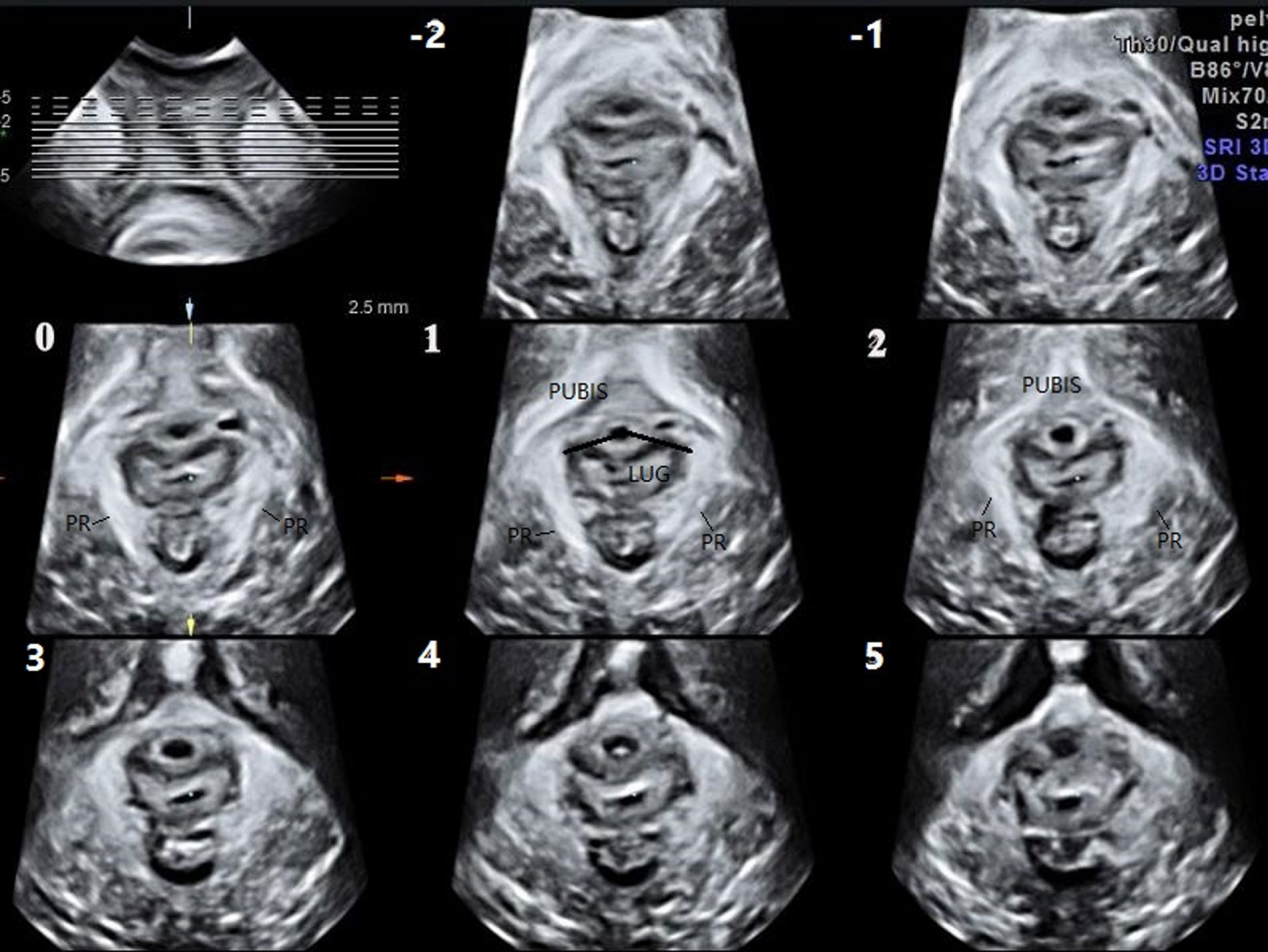
Images of the normal levator ani muscle of a 20-year-old nulliparous woman on TUI are presented. The image in the top left corner is a reference slice in the coronal plane. The other eight images represent axial plane slices obtained at 2.5-mm intervals; slice 1 is obtained in the plane of the minimum levator hiatus; the -1, -2, and 0 slices are caudal of the minimum plane; and the 2, 3, 4, and 5 slices are cephalad of the minimum plane. LUG was measured in slices 0, 1, and 2 and then averaged. (TUI: Tomographic ultrasound imaging; LUG: Levator-urethra gap, PR: puborectalis).

**Figure 2:**
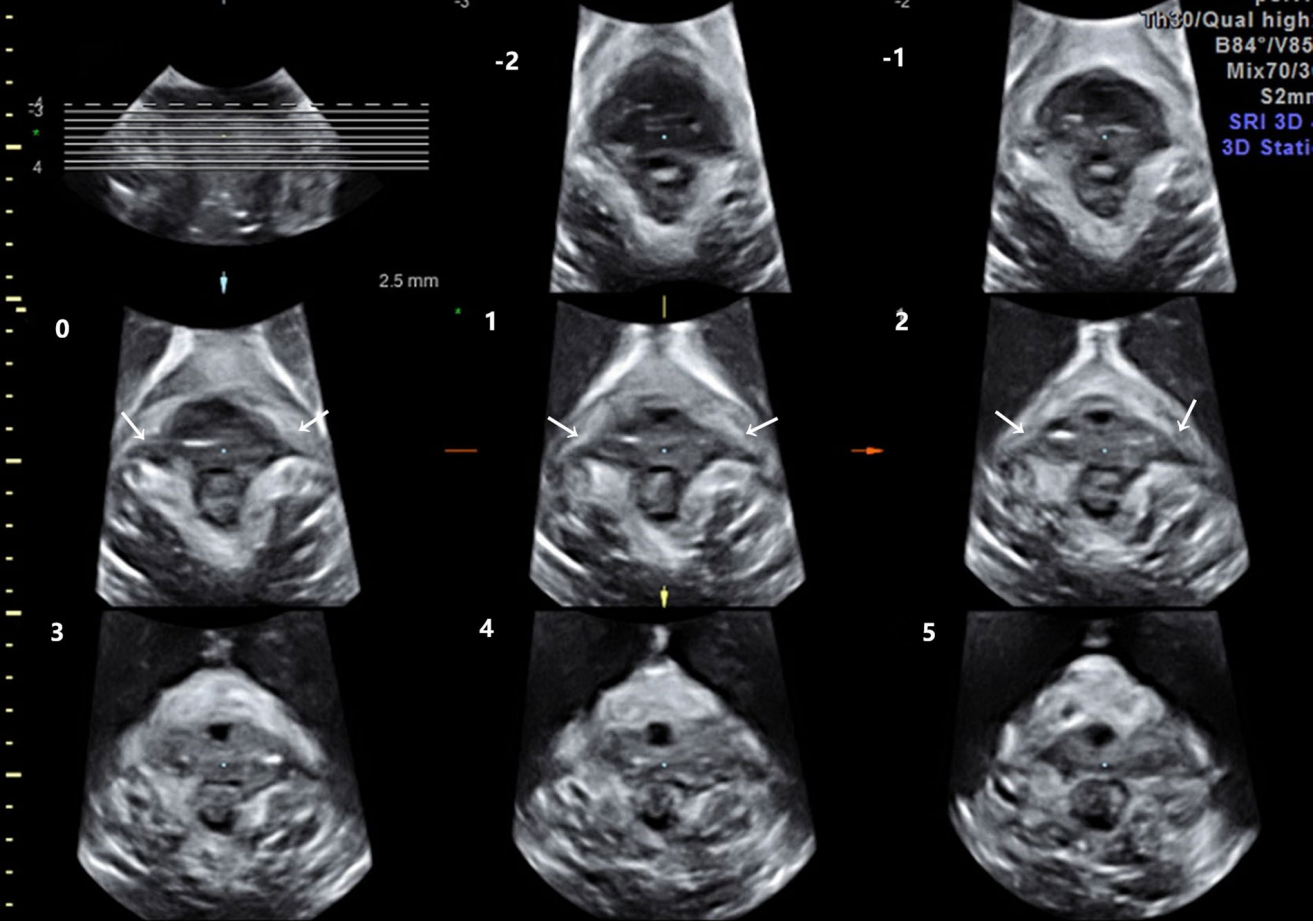
The transperineal views of the puborectalis muscle obtained by TUI show bilateral avulsion, as marked by arrows. There are abnormal echo inserted between the muscle and pelvic sidewall on slice −1 to slice.

No cases of puborectalis avulsion were found in the nulliparae group. The levator hiatus had a compact structure outlined by the pubis and puborectalis. The puborectalis was continuous and formed a V-shaped sling running from the pelvic sidewall towards the anorectal junction. The puborectalis closely attached to the interior edge of the inner surface of the pubic bone (without abnormal echo inserted) and surrounded the posterior rectum on the dorsal side (Fig. 1). Puborectalis avulsion was identified as a loss of continuity between the muscle and the pelvic sidewall, with no muscle remaining on slices 0, 1, and 2 of TUI (Fig. 2). The iliococcygeus is difficult to observe on translabial ultrasound images. In the 80 vaginally parous women, puborectalis avulsion was diagnosed in 11 women (13.75%). Defects were on the left (n = 3), on the right (n = 4) or bilateral (n = 4). Of these 11 cases of puborectalis avulsion, 7 were spontaneous vaginal deliveries, and 4 were forceps-aided vaginal deliveries. Puborectalis avulsion was more common in forceps-aided vaginal delivery than in spontaneous vaginal delivery (χ^2^ = 12.703, P < 0.001). Furthermore, in the primiparae group, the LUG in women with puborectalis avulsion were much larger than that of women with an intact levator (P < 0.001) (Table 2).

**Table 2:** LUG in women with puborectalis avulsion were much larger than women with intact levator in primiparae group (P < 0.001).

Group	LUG (mm)		
	Rest	Contraction	Valsalva
Levator avulsion	27.97 ± 1.88	27.55 ± 2.96	28.36 ± 2.63
Intact levator	19.56 ± 2.68	18.05 ± 2.13	21.19 ± 2.63
*P* values	<0.001	<0.001	<0.001

(LUG: Levator-urethra gap).

### MRI results

**Figure 3:**
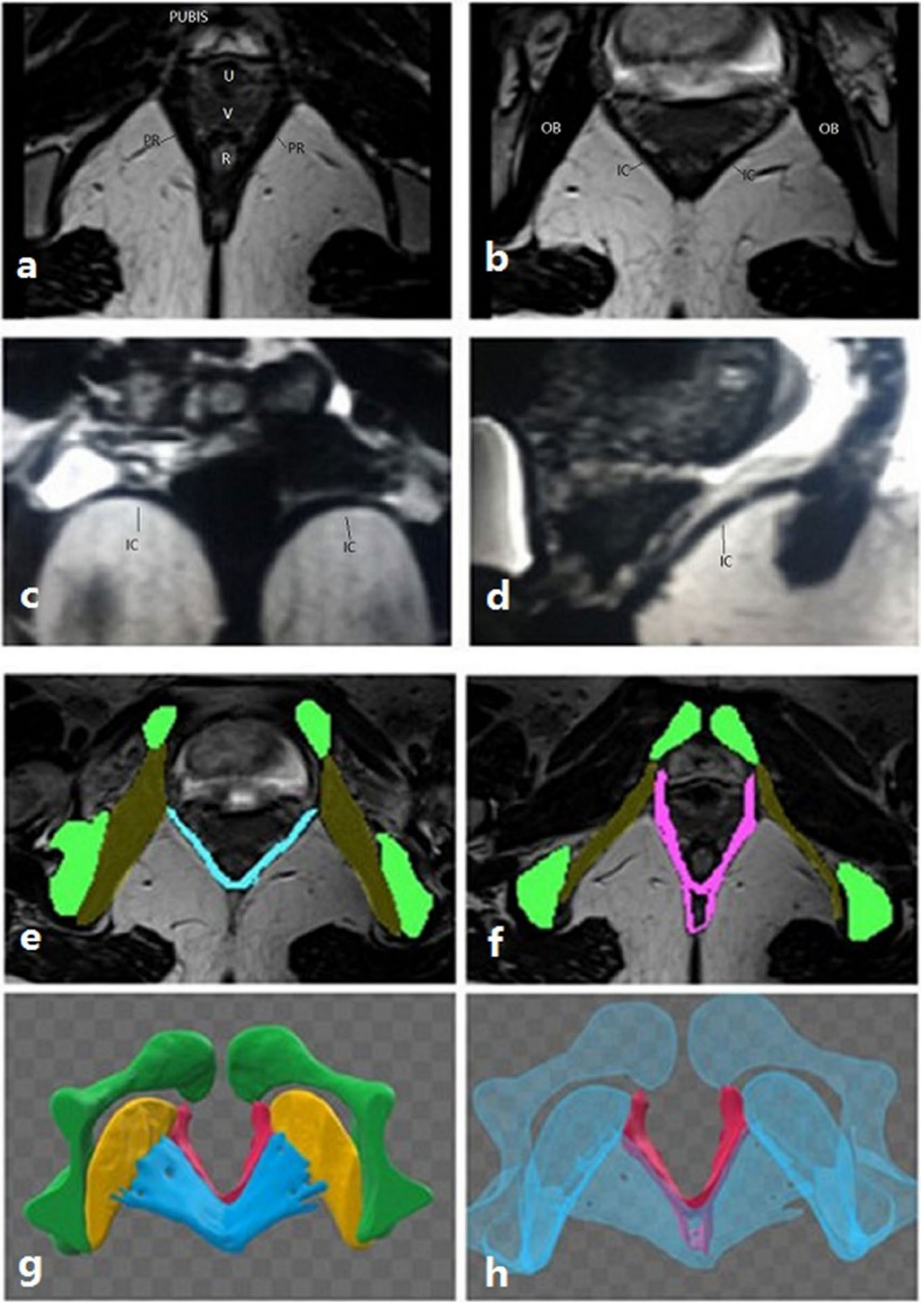
(**a**) Shows that the puborectalis originates from the inner surface of the pubic bone and bypasses the rectum to the inner surface of the pubic bone on the other side, like a sling. (**b–d**) Demonstrate the morphology of the iliococcygeus in the axial, coronal and sagittal planes, respectively. In the coronal plane, it is similar to a wing. In (**e,f**), the pelvic bone structures are presented in green, the obturator muscle in yellow (showing its attachment to the pelvic medial surface), the iliococcygeus muscle in blue and puborectalis in red. (**g,h**) Shows the 3D model of the iliococcygeus and puborectalis. In (**g**), the blue structure is the iliococcygeus, and the red structure is the puborectalis. To better display the puborectalis, which is in the bottom of the iliococcygeus, the iliococcygeus, obturator muscle and bone structure were made transparent as seen on (**h**). (PR: puborectalis, IC: iliococcygeus, OB: obturator muscle).

**Figure 4:**
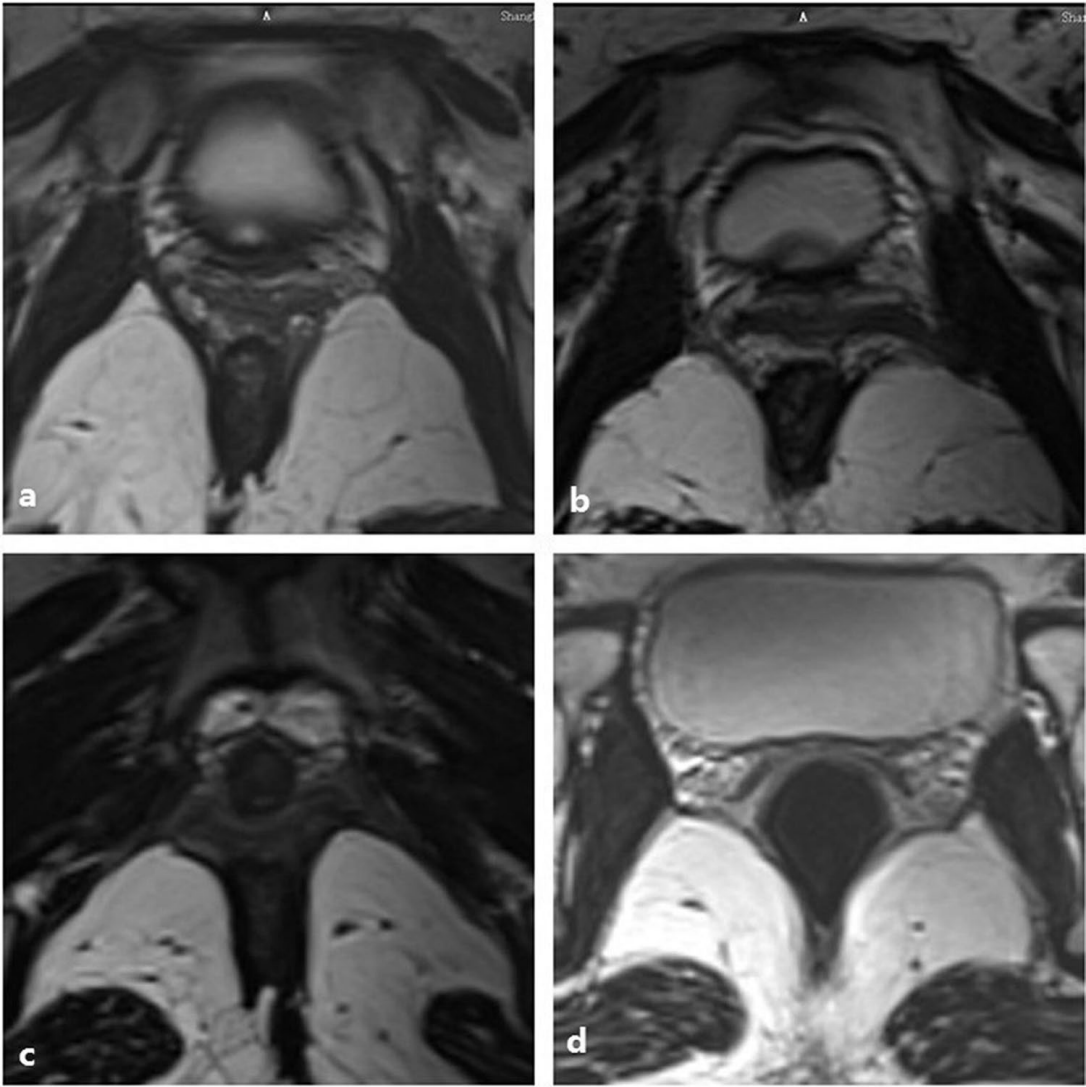
(**a**) Reveals that the left part of the iliococcygeus muscle fibre become thin without obvious avulsion. No defect is found on the right side. (**b**) Shows that the left iliococcygeus muscle became thin and detached from the arcus tendinous levator ani that overlies the obturator internus muscle. (**c**) Shows the left avulsion with the obturator muscle injury and an abnormal signal inserted into their connection. (**d**) Shows that bilateral iliococcygeus muscles became thin. The left side muscle defected in the middle part without fracture, similar to a greenstick fracture.

The levator ani muscle can be distinguished as the puborectalis and iliococcygeus on MRI. Moreover, the levator ani muscle can be observed in the axial, coronal and sagittal planes (Fig. 3). The puborectalis originates from the inner surface of the pubic bone and runs dorsal to the rectum as a sling (Fig. 3a). The MRI results demonstrated that puborectalis avulsions occurred on the left (n = 4), on the right (n = 4) or bilaterally (n = 3). The iliococcygeus originates from the arcus tendinous levator ani (ATLA), which overlies the obturator internus muscle, bypassing the rectum to the ATLA on the other side (Fig. 3b). In the coronal plane, it was similar to a wing (Fig. 3c). When the wing-like morphology disappeared, damage to the iliococcygeus should be assessed. The sagittal images also showed the orientation of the iliococcygeus muscle (Fig. 3d). On the MRIs, the morphological changes of the iliococcygeus could be well-observed (Fig. 4); it became thin but was still attached to the ATLA (n = 6) (Fig. 4a) or deficient in the middle without avulsion (n = 2) (Fig. 4d), detaching from the ATLA with an obturator muscle injury (n = 2) (Fig. 4b) or without an obturator muscle injury (n = 3) (Fig. 4c).

### Comparison between MRI and ultrasound results

**Figure 5:**
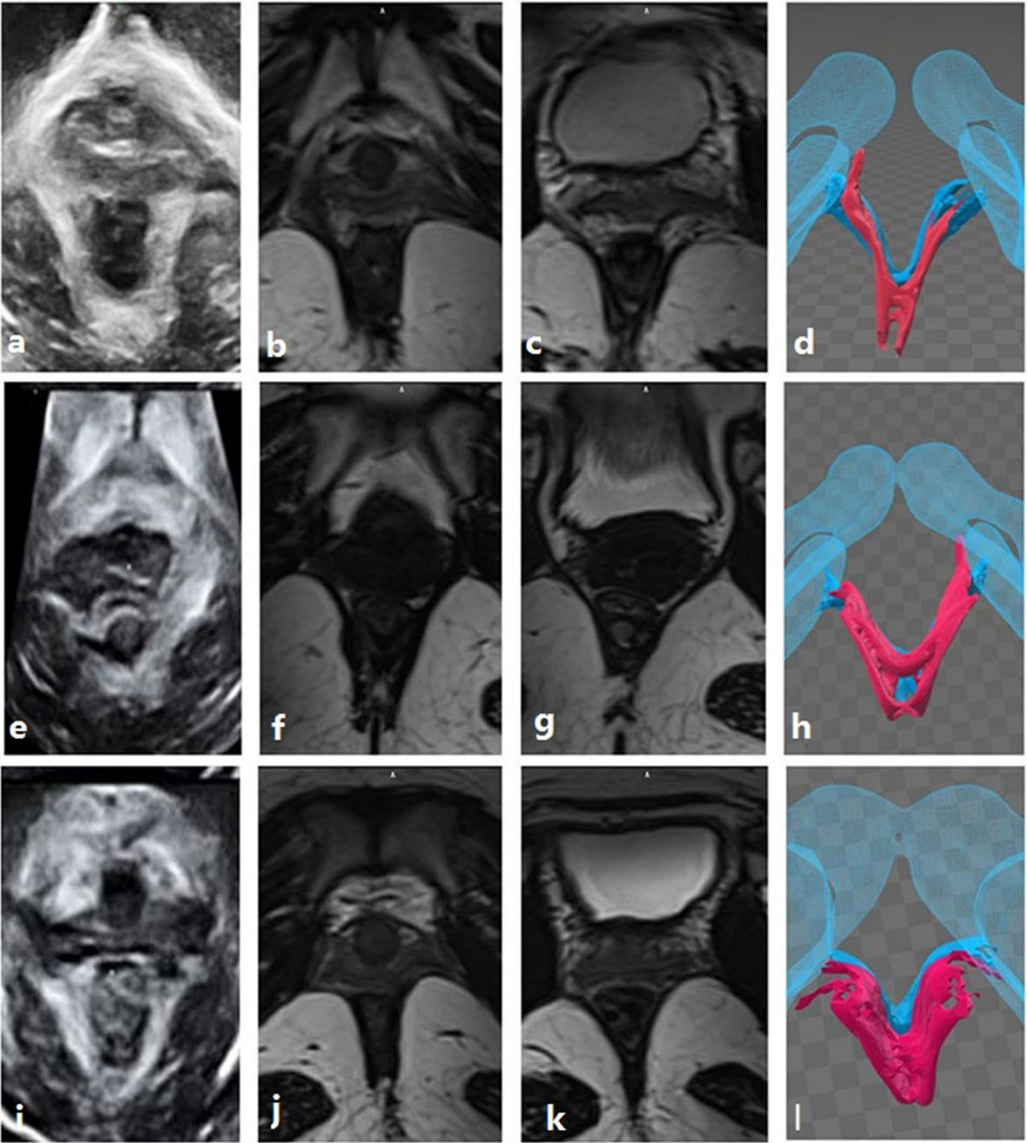
The ultrasound images, MRIs and three-dimensional models of the three cases are shown. (**a–l**) Represent the same cases, respectively. Images in the first column are ultrasound images, images in the second column are MRIs used to observe the puborectalis, and images in the third column are MRIs used to observe the iliococcygeus and 3D model which show a tomographic overview of the puborectalis and iliococcygeus. Images in the first line are the left puborectalis avulsion with left iliococcygeus avulsion. The images in the second line are the right puborectalis avulsion without iliococcygeus damage. The images in the third line are the bilateral puborectalis avulsion with left iliococcygeus avulsion, including the left internal obturator muscle injury.

Puborectalis avulsions were on the left (n = 3), on the right (n = 4) or bilateral (n = 4) on ultrasound images. Similarly, the MRI results demonstrated puborectalis avulsions occurred on the left (n = 4), on the right (n = 4) or bilaterally (n = 3). MRI and ultrasound have good consistency in diagnosing puborectalis avulsion (concordance rate = 90.9%, the kappa coefficient = 0.864) (Table 3) (Fig. 5).

**Table 3:** Comparison of diagnosis of levator avulsion between 3D-US and 3D MRI-based models.

Number	US	MRI	
	puborectalis	puborectalis	iliococcygeus
01	L	B	L (with OB injury)
02	B	B	L (with OB injury)
			R (deficient in the middle part without fracture)
03	B	B	L
04	B	B	L (deficient in the middle part without fracture)
05	L	L	(−)
06	L	L	L
07	L	L	L
08	R	R	(−)
09	R	R	(−)
10	R	R	(−)
11	R	R	(−)

(L: left avulsion; R: right avulsion; B: bilateral avulsion; US: Ultrasound).

## Discussion

The aetiology of pelvic floor dysfunction (PDF) is multifactorial, and among the factors, the integrity of the levator ani muscle is very important. Vaginal delivery is one of the most important factors for levator avulsion, which may destroy the integrity of the levator ani muscle^17^. However, there have been few imaging studies in the published literature comparing the levator ani muscle before and after childbirth using 3D ultrasound and a 3D MRI-based model. Dietz first documented the intrapartum diagnosis of major delivery-related levator trauma in a patient who suffered a large vaginal tear at the time of a normal vaginal delivery in 2007^16^. In this study, to explore the morphological changes of the levator ani muscle (including the puborectalis and iliococcygeus) and levator avulsion after vaginal delivery, TUI, MRI and a 3D MRI-based model were used to examine vaginally parous Chinese women.

Theoretically, there can be different degrees of tears, ranging from the loss of a few fascicles to disruption of all the muscle. However, it can be expected that partial injuries might be compensated by surrounding muscle, but full avulsion may exceed the ability of remaining muscle that cannot replace lost contractile force. Full avulsion is difficult to reverse and there is no other surrounding muscle to compensate, so there is a higher risk of PFD compared to partial injuries^18^. Therefore, in this study, full avulsion of the levator ani muscle was recorded.

Translabial 3D ultrasound is non-invasive and by far the easiest, cheapest and most widely available imaging at present. 2D ultrasound can diagnose levator avulsion by detecting discontinuity between the hyperechogenic fibres of the puborectalis muscle and pelvic sidewall^19^. However, the findings are less repeatable than those found on 3D ultrasound. Rendered volumes and tomographic imaging have been widely used, especially tomographic ultrasound imaging, which is used by many clinicians around the world to diagnose levator avulsion. TUI avoids the disadvantage of rendered volume technique, which has only one slice representation of the levator ani muscle, so it can reduce false-positive results^13^. Zhuang RR observed that patients with PFD showed that TUI and 3D MRI-based modelling used to diagnose puborectalis avulsion had substantial agreement^20^. Our study demonstrated that translabial 3D ultrasound is a good assessment for diagnosing puborectalis muscle avulsion and is consistent with the MRI results. Dietz’s study showed that the measurement of LUG was strongly associated with puborectalis avulsion trauma, and a cut-off value of 25 mm could be used to diagnosis puborectalis avulsion. In our study, the LUG of the avulsion side was 27.55 ± 2.96 mm, mainly consistent with Dietz’s result^21^.

Due to its superior discriminatory capabilities, pelvic MRI has been becoming more popular recently. MRI can show the details of the levator ani muscle and display the different components of the levator ani muscle. Furthermore, a 3D MRI-based model can provide more details about the levator ani muscle. It provides a tomographic overview to observe the stereoscopic levator complex and shows more details of the puborectalis and iliococcygeus muscle at their origin and insertion^22^. The detection of either the minor avulsion or full avulsion using 3D MRI is much more sensitive than 3D ultrasound. In this study, ultrasound images of 11 postpartum women showed left avulsion of the puborectalis muscle in 4 cases, right avulsion in 4 cases, and bilateral avulsion in 3 cases. One of them, who was diagnosed with only left avulsion on ultrasound, showed complete avulsion on both sides on MRI. The consistency of diagnosing puborectalis avulsion between ultrasound and MRI is good. However, it is difficult to observe the iliococcygeus on ultrasound. Due to the wider range of inspection and good soft tissue resolution of MRI, it can complement the lack of ultrasound in this regard. Bilateral iliococcygeus lesions were also recorded separately. The MRI results showed that iliococcygeus muscle injuries could be expressed as becoming thinner (n = 6). The iliococcygeus muscle was separated from the TALA in 5 patients, and injury of the obturator muscle was found in 2 patients. In 2 patients, the iliococcygeus muscle are deficient in the middle part without obvious avulsion, similar to a ‘greenstick fracture’. However, all iliococcygeus muscle injuries occurred on the ipsilateral side of the puborectalis muscle that was gravely damaged. Accordingly, when the puborectalis muscle was not obviously abnormal, the ipsilateral iliococcygeus muscle was also normal. Therefore, in routine clinical work, ultrasound has rapid and convenient advantages to examine postpartum women. However, we recommend that if a patient is found to have a severe injury of the puborectalis, it is necessary to perform an MRI examination to rule out iliococcygeus muscle injuries.

This study was based on a small database. To confirm our results, further study of larger databases is still needed. The number of the cases should increase to include postpartum women with normal puborectalis observed on ultrasound to confirm our results with a larger database.

## Conclusion

Vaginal delivery may result in levator avulsion, including puborectalis avulsion and iliococcygeus avulsion. Both MRI and TUI can detect puborectalis avulsion well, and their results have good consistency. Additionally, MRI can be used to diagnose iliococcygeus avulsion, and iliococcygeus muscle injury is often associated with severe puborectalis muscle tear.

## Materials and Methods

### Subjects

This study was conducted in Shanghai Jiao Tong University Affiliated Sixth People’s Hospital from January 2015 to December 2015. This research was conducted in accordance with the guidelines of the National Institutes of Health of China. All the experimental protocols were approved by the ethics committee affiliated with Shanghai Sixth People’s Hospital. In the prospective observational study, 80 women after first vaginal delivery (primiparae group) and 30 nulliparous women (nulliparae group) were examined with translabial 3D ultrasound and MRI. All the subjects signed the informed consent before being selected for this study. Inclusion criteria for the primiparae group were women who had given birth in our delivery room (45–60 days after delivery) with uncomplicated singleton pregnancy, full-term birth, and cephalic presentation and without complications such as foetal growth restriction, gestational diabetes, hyperemesis gravidarum or hypertension of pregnancy. In the primiparae group, 72 women had spontaneous vaginal deliveries and 8 women had forceps-aided deliveries. The mean age of the primiparae group was 24.32 ± 3.70 years old, and that of the nulliparae group was 23.84 ± 3.85 years old. The mean gravidity of the vaginally parous women was 2.2 (range 1–3). The nulliparae group was also selected from the Department of Gynaecology for the treatment of irregular menstruation or vaginitis and did not have pelvic floor dysfunction, pelvic trauma or surgery. The exclusion criteria for MRI were the presence of an intrauterine device made of metal or claustrophobic tendencies that would preclude undergoing MRI.

### Ultrasound examination

Translabial ultrasound was performed on these women in a supine position after voiding using the GE Voluson E8 system (GE Kretz technik GmbH, Zipf, Austria) with an RAB 4–8 MHz transducer. Volume datasets were acquired at rest on maximum pelvic floor muscle contraction and maximum Valsalva. Each patient performed at least 3 Valsalva manoeuvres, and the best one was used for the evaluation. Volumes obtained on maximum pelvic floor muscle contraction were particularly useful, as defects seemed to become more defined on contraction of the muscle^23^. Using TUI, a set of 8 parallel tomographic slices was obtained in the axial plane at intervals of 2.5 mm from 5.0 mm caudal to 12.5 mm cephalad of the plane of minimum levator hiatus (Fig. 1). Puborectalis avulsion was identified at the plane slices 0, 1, and 2 on maximum pelvic floor muscle contraction^21^. Avulsion was recorded as left, right or bilateral. In this study, avulsion was defined as no muscle remaining on slices 0, 1, 2 of TUI. The measurement of the levator-urethra gap (LUG) was taken by placing callipers on the centre of the hypoechogenic structure that indicated the urethral mucosa and smooth muscle and on the most medial aspect of the muscle insertion. LUG was measured in three slices and then averaged. The images were analysed separately by 2 senior authors with abundant experience in diagnosing levator avulsion, but they were blinded to each other. If different results occurred, they consulted to make a decision.

### MRI examination

MRI was performed in one week if puborectalis avulsion was found on at least one side on TUI. The examination was performed using a 3 T scanner (T2M Trio, Siemens, Erlangen, Germany). An external phased-array body coil was employed and was centred at the lower pelvis in supine position. Before examination, all subjects were asked to empty their bladder. Due to the difficulty of holding the Valsalva strain or contract effort during MRI scan, we only analysed static images in this study. Static native T2-weighted turbo spin echo sequences were acquired in the axial plane (TR 1260 ms, TE 130 ms, FOV 400 mm, Slice Thickness 1.00 mm). Section orientation of the axial plane was parallel to the horizontal line—specifically, the levator hiatus—defined as a straight line from the inferior margin of the pubic symphysis to the posterior part of the puborectalis sling^24^. The morphologies of the puborectalis and iliococcygeus are shown in Fig. 3, and whether there is avulsion in the axial plane, coronal plane and sagittal plane can be defined respectively. The images were analysed by 2 senior radiologists who were blinded to the ultrasound results. Subsequently, the axial plane images were imported into Mimics imaging software to create a 3D model. A 3D model presented the pubic bone, puborectalis, iliococcygeus and internal obturator muscle by tracing the structure outlines on axial images. All 2D images were used to make a 3D model to detect the levator avulsion, including the puborectalis and iliococcygeus avulsion (Fig. 3).

### Statistical analysis

A statistical analysis was performed using the Statistical Analysis System 8.0 statistical program (SAS Institute Inc., Cary, NC, USA). The measured parameters were presented as the mean positive and negative standard deviation ($\bar{\chi }$ ±s). The LUG between the various groups was compared using an independent sample t-test. The χ^2^ test was used to test the difference in levator injury between spontaneous vaginal delivery and forceps-aided delivery and the results of the TUI and MRI. A value of P < 0.05 was considered statistically significant.
